# Association between weight loss, change in physical activity, and change in quality of life following a corporately sponsored, online weight loss program

**DOI:** 10.1186/s12889-022-12835-4

**Published:** 2022-03-07

**Authors:** Christoph Höchsmann, James L. Dorling, Corby K. Martin, Conrad P. Earnest, Timothy S. Church

**Affiliations:** 1 Pennington Biomedical Research Center, Baton Rouge, LA USA; 2 Department of Sport and Health Sciences, Technical University of Munich, Connollystraße 32, Munich, 80809 Germany; 3 Human Nutrition, School of Medicine, Dentistry and Nursing, College of Medical, Veterinary and Life of Sciences, University of Glasgow, Glasgow, UK; 4 Texas A&M University, College Station, TX USA; 5 Wondr Health™, Dallas, TX USA

**Keywords:** Quality of life, Weight loss program, Corporate health, Web-based, Online, Physical activity

## Abstract

**Background:**

The physiological benefits associated with corporately sponsored weight loss programs are increasingly well documented. However, less is known about how these programs affect employees’ quality of life (QoL). The purpose of the present analysis was to examine the association between weight loss, change in physical activity, and change in QoL following a corporately sponsored, online weight loss program.

**Methods:**

We examined the relationship between weight loss, self-reported change in physical activity, and change in several QoL indices in 26,658 participants (79% women) after the initial 10 weeks of the online weight loss program. The trend in changes in each QoL index with increasing weight loss and change in physical activity was examined using logistic regression analysis.

**Results:**

We observed greater improvements in each QoL index with increasing weight loss (*p*-for-trend, < 0.001) as well as with progressive increases in physical activity (*p*-for-trend, < 0.001). The combination of increasing weight loss and increases in physical activity were associated with the greatest improvements in each QoL index (additive effect). The percentage of employees reporting improvements in QoL (“improved” or “very much improved”) was 64% for energy, 63% for mood, 33% for sleep, 65% for self-confidence, 68% for indigestion, and 39% for musculoskeletal pain.

**Conclusions:**

Among people, who engage with a commercial weight loss program, greater weight loss during the program was associated with greater improvements in QoL, and increases in physical activity further enhanced the QoL-related benefits.

## Background

Obesity continues to be a highly prevalent disease that directly affects health, health care costs to the individual and employers, overall psychological health, and quality of life (QoL) [1]. It is now estimated that 49% of American adults have attempted to lose weight, often turning to commercial programs to help them in their quest [2]. Within this context, several commercial programs are available online through membership subscriptions to consumers [3]. Workplace curricula also continue to emerge by offering programs directly to individuals to decrease the corporate burden of health care costs [4]. The utility of such programs has been reported in a 2016 systematic review by Michaud et al. [4], as well as continued reports by our group and others [5–11]. Although the physiological benefits associated with such programs are increasingly well documented, less is known about how these programs affect employees’ QoL.

In general, as shown by the National Health and Wellness Survey (*N* = 70,000), a higher body mass index (BMI) is associated with lower QoL and reduced work productivity [12]. At the same time, previous research within different settings and populations has demonstrated the relationship between weight loss and improvements in QoL. For example, Hageman et al. found that rural women who lost ≥10% of weight during a web-based lifestyle intervention over 18 months showed significantly greater improvements in QoL domains such as depression, physical function, pain interference, fatigue, and satisfaction with social role compared to women who gained weight or did not achieve clinically significant weight loss (≥5%) [13]. Further, significant improvements in the QoL domains of physical function, self-esteem, sexual life, and work, as well as in depression and perceived stress have been reported after a 14-week intensive lifestyle intervention (in-person) in adults with obesity, with greater weight loss being associated with greater QoL-related improvements [14]. Importantly, the improvements in QoL in that study were largely maintained at the 52-week follow-up despite partial weight regain. It has also been shown that adding either aerobic or resistance training to a 6-month dietary weight loss program elicits significantly greater improvements in QoL than the dietary weight loss component alone [15], highlighting the importance of physical activity (PA) to improve QoL during behavioral lifestyle interventions. In the workplace setting, Clark et al. found that employees who used a worksite wellness center (program offers included aerobic fitness, healthy nutrition, weight management, musculoskeletal conditioning, and stress reduction) 2–3 times per week showed greater improvements in QoL after 1 year compared to employees who used the wellness center only once every 2 weeks [16]. Finally, even very brief behavioral programs can be effective in improving participants’ well-being as Das et al. have demonstrated in their randomized controlled trial (12 diverse worksites, *N* = 240), showing improvements in vitality, sleep, and overall QoL 6 months after completion of the trial’s intensive 2.5-day group-based behavioral intervention [17]. Collectively, these findings suggest that a web-based, behavioral weight loss program has promise to elicit meaningful improvements in QoL, and the worksite provides an ideal setting to implement such programming. A majority of adults spend substantial time at work and employers’ interest in providing wellness programs to their employees has increased in recent years due to the rapidly rising cost of providing healthcare for employees to treat obesity and obesity-related diseases that are impacting QoL [18, 19].

In the primary analyses reported herein, we examined the relationship between weight loss and change in several QoL indices in a large sample (*N* = 26,658) following participation in a corporately sponsored, online, behavioral weight loss program over 10 weeks. In secondary analyses, we examined the relationship between weight loss, QoL indices, and self-reported changes in PA. While PA is not the primary focus of the program, regular moderate-intensity PA is advocated within the program. We hypothesized that weight loss would be positively associated with improvements in each QoL index and that increases in PA during the program would further enhance these QoL improvements.

## Methods

### Participants

The present report describes the examination of 26,658 individuals (79% women, BMI ≥25.0) participating in an online, behaviorally oriented, commercial weight loss program for company employees from various states within the United States. Companies contacted their employees regarding participation in the online weight loss program via emails and flyers placed at the worksites, and participation was voluntary. The focus of the current analyses is on the relationship between weight loss, categories of change in PA, and indices of QoL. The study was reviewed by an ethics committee (Chesapeake IRB, Columbia, MD). Because the research presented in the manuscript is secondary data analysis of de-identified data, the requirement to document informed consent was waived and the study was determined to be exempt from IRB oversight according to the tenets of the US Department of Health and Human Services regulations at 45 CFR 46.

### Program curriculum

All participants were enrolled in the online weight loss program (Wondr Health™, formerly Naturally Slim, Dallas, TX, USA) through their respective employers. The yearlong program is composed of 10 weekly classes, followed by seven bi-weekly and six monthly maintenance classes for a total of 52 weeks. Classes are based on Specific, Measurable, Attainable, Realistic, Time-based (SMART) behavioral goal-setting practices [20, 21], and the program curriculum focuses on specific elements found in standard behavioral weight management programs such as self-monitoring, goal setting, stimulus control, modification of eating habits, and problem-solving while concentrating on mindful healthy eating and understanding hunger signals. The focus of each of the weekly classes is as follows: (1) Mindful Eating and Portion Control, Stimulus Control, Medical Considerations & Weight Loss, (2) Stop Eating Cues, Introduction to PA, (3) Stress and Emotions, Mindless Eating, Goal Setting and Problem Solving, PA, (4) Hidden Sugar, Mindful Activities, Energy Balance, (5) Nutrition 101, Stress Management, PA & Weight Maintenance, (6) Weight Fluctuations, Food Cravings vs. Easily Accessible Food, Centers for Disease Control and Prevention (CDC) Exercise Recommendations, (7) Emotions and Eating, Importance of Self-Monitoring, Making Exercise A Habit, (8) Grocery Shopping and Meal-Planning, Metabolic Syndrome, Cognitive Behavioral Techniques, (9) Serving Sizes, Social Support, Dealing with Saboteurs and (10) Review of Eating Skills and Tools, Maintaining Motivation, and Long-Term Action Planning. An outline of all program objectives has been previously published [9]. Participants are encouraged to reduce intake of carbohydrates and sugar, particularly refined sugars, and to maintain a protein intake of 25–30% of total calories. The program does not place a special focus on eliminating specific food groups or macronutrients from participants’ diets, however. Participants are further encouraged to engage in moderate-intensity PA, primarily walking, per the NIH consensus development panel on PA and cardiovascular health [22]. Program classes are distributed via a web-based distance-learning platform, allowing participants to attend anywhere and anytime at their convenience, given access to the Internet.

### Outcome measures

All outcome data reported herein were obtained from participants’ self-report, recorded online, and stored in a central database.

#### Change in quality of life

At the 10th week of the program, changes in six indices of QoL (energy, mood, sleep, self-confidence, indigestion, and musculoskeletal pain) were assessed via questions asking, “How has your *QoL index 1-6* changed compared to before starting the online weight loss program?” with response options being “very much improved”, “improved”, “no change”, and “worsened”.

#### Change in physical Activity

Similar to QoL, changes in PA were assessed upon completion of the program via a 5-point Likert scale (“quite a bit more”, “slightly more”, “no change”, “slightly less”, and “much less”).

#### Weight change

Total weight change in kg and percent weight change was calculated based on the self-reported weights before the start and after completion of the program, as post-program weight – pre-program weight and (post-program weight – pre-program weight) / pre-program weight × 100.

### Statistical analyses

The present analysis examines findings relative to participation in the weight loss program, and we included participants who attended ≥8 of the first 10 weekly classes (26,658 of 27,814 participants who completed the QoL survey; 96%). The primary outcomes were indices reflecting QoL defined as changes in (1) energy, (2) mood, (3) sleep, (4) self-confidence, and clinical QoL indices (5) indigestion and (6) musculoskeletal pain. The descriptive characteristics of the study sample were examined for all participants as well as by gender. Continuous data are presented as mean (standard deviation [SD]) and categorical data as N (%). Weight change (in kg and percent) is presented with corresponding 95% confidence intervals (CI).

Using logistic regression (proc logistic), change in QoL (binary) was regressed against percent weight loss (continuous) and change in PA category with adjustment for age and gender. To create the binary QoL variable, the four QoL categories were collapsed into the two categories “improved”, composed of “very much improved” and “improved”, and “not improved” composed of “no change” and “worsened”. Weight loss was categorized using the clinical cut-points < 3.0%, 3.0–4.9, and > 5.0%. For the change in PA category, due to the very small number of participants in the “slightly less” (1.4%) and “much less” (0.3%) PA categories, we collapsed these groups into the “no change” category, resulting in the three PA change categories “quite a bit more”, “slightly more”, and “no change”. Using a generalized linear model adjusted for age and gender, we examined the percentage of participants indicating improvements in the binary QoL category (“very much improved” or “improved”) for all nine combinations of weight loss category and change in PA category.

All analyses were performed with SAS version 9.4 (SAS Institute Inc., Cary, NC), and significance was accepted at *p* < 0.05.

## Results

In total, 388,600 employees started the corporately sponsored weight loss program and 174,821 participants (45%) attended ≥8 classes. Of those who attended ≥8 classes, 26,658 completed the QoL survey and they are included in the present analyses (Table 1**)**. Participants (79% women) were 53.8 (SD 9.8) years old, attended 9.9 (SD 0.3) of the total of ten classes, and lost a significant amount of their starting weight (− 4.6% [95% CI, − 4.61, − 4.56]). Participants who attended ≥8 classes but did not complete the QoL survey (*n* = 148,163; 74% women; mean age: 48 years; mean pre-program weight: 96.1 kg; mean weight loss: 3.4%) were excluded from the analyses presented herein.

**Table 1:** Characteristics of study participants

	**All (*N*** **= 26,658)**		**Women (*n*** **= 20,992)**		**Men (*n*** **= 5666)**	
	**Mean**	**(SD)**	**Mean**	**(SD)**	**Mean**	**(SD)**
Age (y)	53.8	(9.8)	53.6	(10.2)	54.5	(9.6)
Classes attended ^a^	9.9	(0.3)	9.9	(0.3)	9.9	(0.3)
Pre-program weight (kg)	93.5	(19.4)	90.6	(18.7)	104.4	(17.9)
Post-program weight (kg)	89.2	(18.8)	86.6	(18.3)	98.8	(17.5)
Weight change (kg)	−4.3	(3.2)	−4.0	(3.0)	−5.5	(3.6)
	[95% CI −4.35, − 4.26]		[95% CI − 4.01, −3.93]		[95% CI − 5.60, − 5.45]	
Weight change (%)	−4.6	(3.3)	−4.4	(3.3)	−5.3	(3.4)
	[95% CI −4.61, − 4.56]		[95% CI − 4.46, − 4.37]		[95% CI −5.41, − 5.23]	
	**N**	**(%)**	**N**	**(%)**	**N**	**(%)**
Change in Weight						
< 3.0%	9319	(35.0)	7742	(36.9)	1577	(27.8)
3.0–4.9%	6535	(24.5)	5236	(24.9)	1299	(22.9)
≥ 5.0%	10,804	(40.5)	8014	(38.2)	2790	(49.2)
Change in Energy						
Very much improved	1959	(7.4)	1565	(7.5)	394	(7.0)
Improved	15,006	(56.3)	11,687	(55.7)	3319	(58.6)
No change	9433	(35.4)	7528	(35.8)	1905	(33.6)
Worsened	260	(1.0)	212	(1.0)	48	(0.9)
Change in Mood						
Very much improved	2121	(8.0)	1731	(8.3)	390	(6.9)
Improved	14,675	(55.1)	11,588	(55.2)	3087	(54.5)
No change	9509	(35.7)	7388	(35.2)	2121	(37.4)
Worsened	353	(1.3)	285	(1.4)	68	(1.2)
Change in Sleep						
Very much improved	839	(3.2)	682	(3.2)	157	(2.8)
Improved	7939	(29.8)	6293	(30.0)	1646	(29.0)
No change	17,341	(65.1)	13,584	(64.7)	3757	(66.3)
Worsened	539	(2.0)	433	(2.1)	106	(1.9)
Change in Self-Confidence						
Very much improved	2648	(9.9)	2160	(10.3)	488	(8.6)
Improved	14,644	(54.9)	11,546	(55.0)	3098	(54.7)
No change	9082	(34.1)	7046	(33.6)	2036	(35.9)
Worsened	284	(1.1)	240	(1.1)	44	(0.8)
Change in Indigestion ^b^						
Very much improved	1124	(16.2)	893	(15.2)	231	(21.6)
Improved	3584	(51.7)	3062	(52.2)	522	(48.8)
No change	2128	(30.7)	1816	(31.0)	312	(29.2)
Worsened	96	(1.4)	91	(1.6)	5	(0.5)
Change in MSK Pain ^c^						
Very much improved	292	(3.4)	222	(3.2)	70	(4.7)
Improved	3060	(36.0)	2473	(35.3)	587	(39.5)
No change	4970	(58.5)	4165	(59.4)	805	(54.1)
Worsened	176	(2.1)	150	(1.8)	26	(1.8)
Change in PA						
Quite a bit more	4383	(16.4)	3400	(16.2)	983	(17.4)
Slightly more	14,290	(53.6)	11,331	(54.0)	2959	(52.2)
No change	7526	(28.3)	5891	(28.1)	1635	(28.9)
Slightly less	368	(1.4)	296	(1.4)	72	(1.3)
Much less	91	(0.3)	74	(0.4)	17	(0.3)

Data are mean (SD) and N (%) unless stated otherwise

^a^ The program consisted of 10 weekly classes, and participants who completed ≥8 classes were included in the analyses

^b^ 6932 participants (5862 women, 1070 men) reported indigestion at baseline

^c^ 8498 participants (7010 women, 1488 men) reported musculoskeletal pain at baseline

Abbreviations: *MSK* musculoskeletal, *PA* physical activity; *SD* standard deviation

Overall, 64% of participants reported more energy (56% “improved” and 7% “very much improved”), 63% percent reported an improvement in mood, (55% “improved” and 8% “very much improved”), 33% reported improved sleep quality (30% “improved” and 3% “very much improved”), and 65% reported an improvement in self-confidence (55% “improved” and 10% “very much improved”). Further, of the 6932 participants who reported indigestion at baseline, 68% showed improvements (52% “improved” and 16% “very much improved”), and, of the 8498 participants who reported having musculoskeletal pain at baseline, 39% reported improvements, (36% “improved” and 3% “very much improved”). Specific gender characteristics of these findings are reported in Table 1. Patterns of changes in QoL indices were similar for women and men.

The majority of participants also reported increases in PA during the program. Fifty-four percent of the entire cohort reported “slightly more” PA and 16% reported “quite a bit more” PA. Approximately 30% reported no change and less than 2% reported decreases in PA throughout the program (Table 1).

**Fig. 1:**
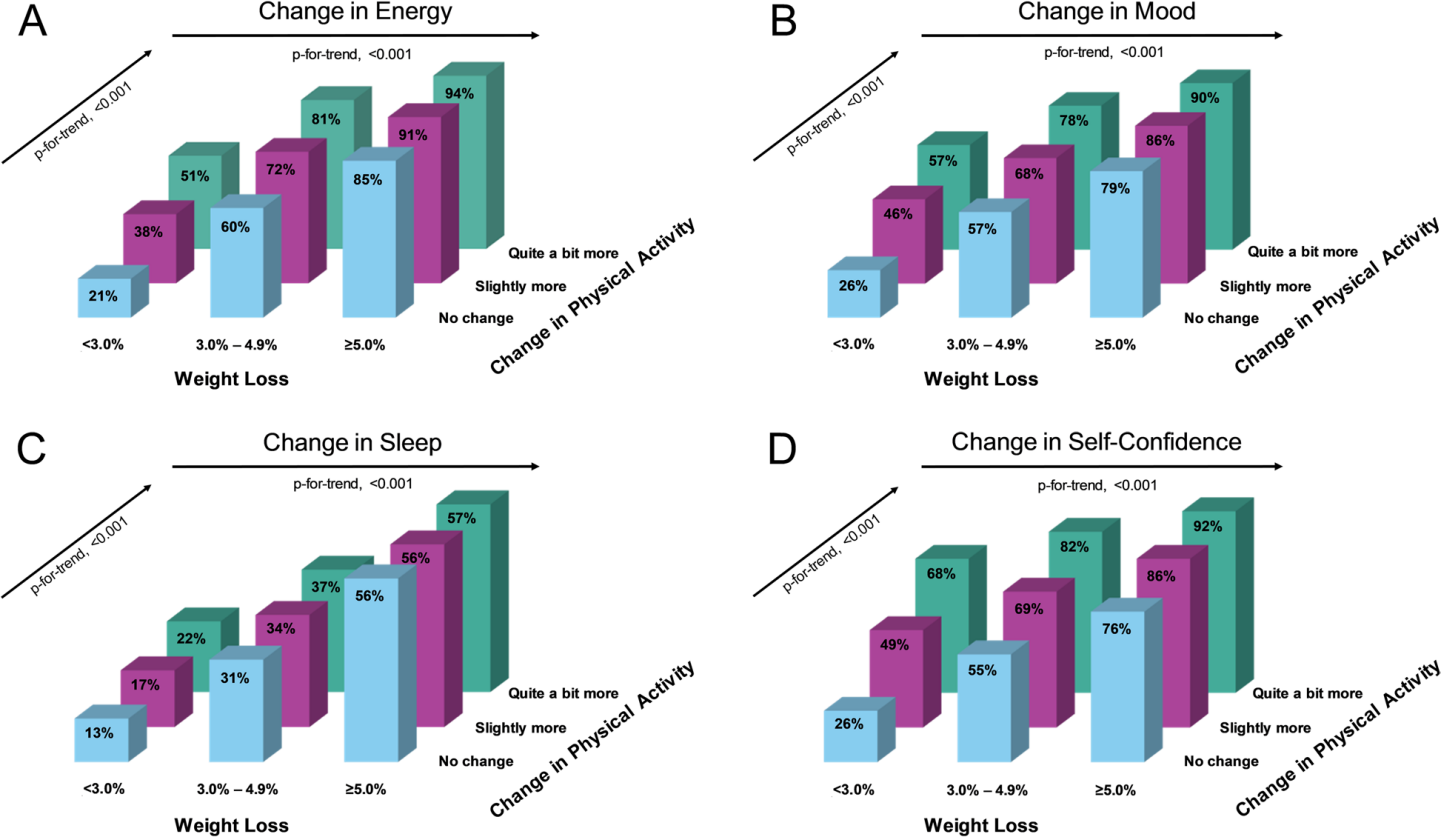
Illustration of the percentage of participants reporting improvements in the personal quality of life indices “Energy” (**A**), “Mood” (**B**), “Sleep” (**C**), and “Self-Confidence” (**D**), relative to percent weight loss category and change in physical activity category

**Fig. 2:**
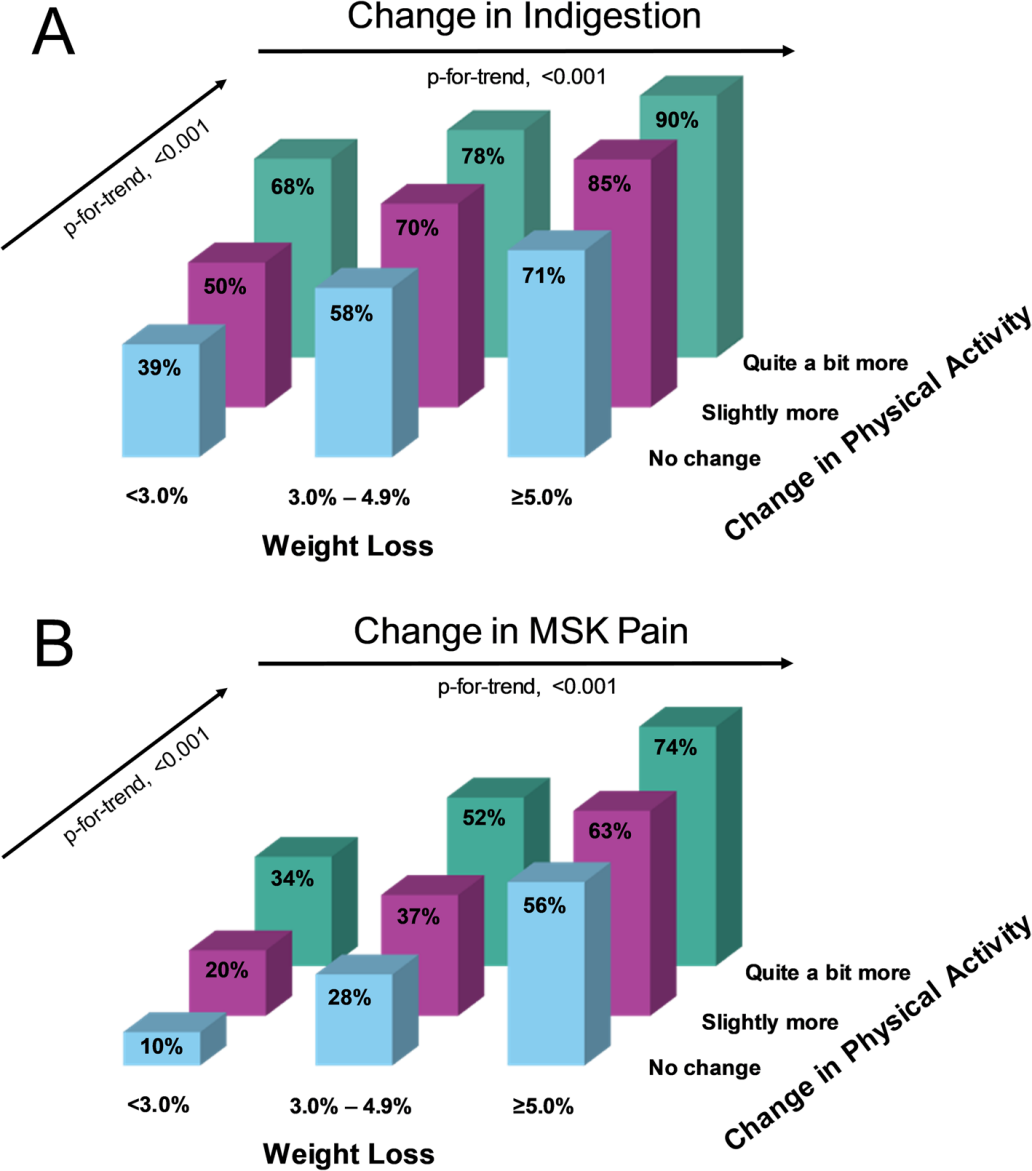
Illustration of the percentage of participants reporting improvements in the clinical quality of life indices “Indigestion” (**A**) and “MSK Pain” (**B**), relative to percent weight loss category and change in physical activity category. MSK, musculoskeletal

When examining the individual QoL indices, we observed a greater percentage of participants who reported improvements in each QoL index with increasing weight loss and with progressing increases in PA. Similarly, the logistic regression analyses (Table 2) showed greater improvements in all QoL indices with increasing weight loss (all *p* < 0.001) and progressing increases in PA (all *p* < 0.001). Each 1% weight loss was associated with an 18% probability of improved energy, 16% probability of improved mood, 4% for sleep, 23% for self-confidence, and 7% for both indigestion and musculoskeletal pain. Similarly, each category increase in PA was associated with a 148% increased probability of improved energy, 106% probability of improved mood, 89% for sleep, 94% for self-confidence, 72% for indigestion, and 95% for musculoskeletal pain. The combination of increasing weight loss and increases in physical activity were associated with the greatest improvements in each QoL index (additive effect). Specifically, the stratum of participants who lost the least amount of weight (< 3.0%) and reported no change in PA had the smallest percentage of participants with improvements in each QoL index. Conversely, the stratum with participants who lost the most amount of weight (≥5.0%) and reported having increased their PA “quite a bit more”, had the greatest percentage of participants with improvements in each QoL index (Figs. 1 & 2).

**Table 2:** Logistic regression analysis for the association between percent weight change and change in physical activity and changes in six indices of quality of life

	B	SE	χ^**2**^	***p***
*Change in Energy*				
Change in Weight (%)	−0.18	0.01	1236.40	**< 0.001**
Change in PA	1.48	0.03	3389.06	**< 0.001**
Age	0.00	0.00	0.05	0.832
Gender	0.02	0.04	0.30	0.582
*Change in Mood*				
Change in Weight (%)	−0.16	0.00	1187.50	**< 0.001**
Change in PA	1.06	0.02	2117.67	**< 0.001**
Age	−0.00	0.00	5.24	**0.022**
Gender	0.25	0.03	54.10	**< 0.001**
*Change in Sleep*				
Change in Weight (%)	−0.04	0.00	90.43	**< 0.001**
Change in PA	0.89	0.02	1671.09	**< 0.001**
Age	−0.00	0.00	1.12	0.290
Gender	0.11	0.03	11.12	**0.001**
*Change in Self-Confidence*				
Change in Weight (%)	−0.23	0.01	1952.26	**< 0.001**
Change in PA	0.94	0.02	1628.96	**< 0.001**
Age	−0.01	0.00	21.64	**< 0.001**
Gender	0.30	0.03	74.84	**< 0.001**
*Change in Indigestion*				
Change in Weight (%)	−0.07	0.00	251.98	**< 0.001**
Change in PA	0.72	0.04	263.09	**< 0.001**
Age	−0.01	0.00	3.31	0.069
Gender	0.16	0.08	4.24	**0.040**
*Change in MSK Pain*				
Change in Weight (%)	−0.07	0.00	420.16	**< 0.001**
Change in PA	0.95	0.04	581.45	**< 0.001**
Age	−0.00	0.00	3.09	0.079
Gender	0.05	0.07	0.60	0.440

Models were adjusted for age and gender. Bold font indicates statistical significance (*p* < 0.05)

Abbreviations: *MSK* musculoskeletal, *PA* physical activity

## Discussion

In the present study, we examined the associations of weight loss and changes in PA levels during participation in a corporately sponsored, online behavioral weight loss program over 10 weeks with various indices related to QoL. In summary, among people who engaged with a commercial weight loss program, greater percent weight loss was associated with a greater percentage of participants reporting improvements in all QoL indices, regardless of changes in PA level. Similarly, greater increases in PA were associated with a greater percentage of participants reporting improvements in all QoL indices, irrespective of percent weight loss. Greater weight loss combined with increases in PA demonstrated an additive effect with regard to improvements in QoL. Therefore, we accept our hypothesis that weight loss will be positively related to improvements in QoL and that increases in PA, as advocated during the program, will further enhance these QoL improvements.

Our results are in line with previous reports showing improvements in mental and physical QoL following participation in a web-based weight loss intervention in the clinical setting [13], as well as at the workplace [13, 23]. Similar to Hageman et al., who reported a comparable mean weight loss of 4.5% following their web-based lifestyle intervention (focus on healthy eating and PA), we found greater weight loss during the program to be positively associated with the percentage of participants reporting improvements in both mental and physical QoL indices [13]. Interestingly, Hageman’s and our findings differ from outcomes of systematic reviews and meta-analyses, which suggest significant positive associations of weight loss with improvements in physical QoL but only minimal or no associations with social or mental QoL indices [24–26]. These differing findings may be explained by the fact that the systematic reviews included a large number of studies that assessed changes in QoL following bariatric surgery rather than following lifestyle modification programs only. It is conceivable that bariatric surgery-related weight loss affects changes in QoL differently than weight loss following a behavioral lifestyle intervention such as ours and that of Hageman et al. [13]. Nevertheless, as shown by van Gemert et al., modest weight loss (~ 6.5%) is not guaranteed to result in QoL improvements even following behavioral lifestyle interventions [27], and the significant variability in QoL after weight loss, that has also been found in a 2017 systematic review of reviews [24], underlines QoL’s complex and multifactorial nature. It further needs to be noted that the aforementioned studies [13, 23–27] assessed QoL with various instruments (Patient-Reported Outcomes Measurement Information System [PROMIS]-29, 36-Item Short-Form Health Survey [SF-36], Impact of Weight on Quality of Life [IWQOL]-Lite, among others). This makes a direct comparison of the changes in QoL among these studies and with our study difficult.

An important finding of our study is that both increasing percent weight loss and greater increases in PA were independently associated with a progressively increasing number of participants reporting improvements in all QoL indices. While PA is not the primary focus of the program, increases are encouraged, and almost 70% of participants reported to have followed the recommendation to increase PA, enhancing the positive effect of weight loss on QoL across all categories of weight loss. These findings are supported by previous results of a randomized controlled weight loss trial by Fanning et al. [15], showing that the addition of either aerobic or resistance training to a 6-month dietary weight loss program elicited significantly greater improvements in QoL than the dietary weight loss component alone and that these improvements were maintained at the 18-month follow-up. Our findings extend those of Fanning et al., who assessed changes in physical health-related QoL only, as they demonstrate weight loss- and PA increase-related improvements in QoL indices that reflect both physical and mental health. Further, the Dose-Response to Exercise in postmenopausal Women (DREW) study [28] found a positive dose-response relationship between the amount of exercise performed and improvements in both physical and mental QoL, supporting the trend of greater increases in PA being associated with greater improvements in QoL, as reported in our study. Similar to our results, in DREW, improvements in QoL were independent of weight loss, demonstrating that increases in PA can lead to significant benefits related to QoL even in the absence of substantial weight loss. Nevertheless, our results also show that the combination of greater weight loss and greater increases in PA acts additively and progressively leads to a greater percentage of participants reporting improvements in all QoL indices.

The assessment of changes in QoL and PA after completion of the program, without baseline assessment, and the lack of control group make it difficult to unequivocally infer causality regarding the relationship between weight loss or changes in PA and improvements in QoL indices. However, a study using multiple mediation analyses to explore the causal mechanisms between weight loss and improvements in QoL in two randomized, placebo-controlled weight loss drug trials has recently demonstrated that improvements in QoL are primarily mediated by weight loss but that decreased depressive symptoms also account for improvements in QoL [29]. In another analysis, the interactive effect of weight loss and treatment group was found to partially mediate the effect of treatment on QoL (more program-related weight loss resulting in improved QoL) during a 12-month behavioral obesity treatment program (focus on diet and daily PA), despite no significant direct effects of treatment on QoL [30]. While the authors found reciprocal effects (i.e., the variables are concurrently mediators and outcomes of treatment) only between weight loss and body image but not between weight loss and QoL, it is generally conceivable that both weight loss and improved QoL during a weight loss intervention are beneficial for program adherence and maintenance of learned behavior changes related to diet and PA and thereby promote further weight loss and concurrent improvements in QoL. So, we can speculate that those participants with more weight loss, greater increases in PA, and greater improvements in QoL, are more likely to maintain the acquired changes in behavior and will consequently be more successful during long-term weight loss and weight maintenance. Future programs should incorporate follow-up assessments to examine these long-term effects.

### Strengths and limitations

A strength of our study is that we examined QoL changes following a corporately sponsored, behavioral weight loss program, using web-based delivery in a large cohort of almost 27,000 participants. We showed a consistent pattern for improved QoL with increasing weight loss as well as with progressive increases in PA during the weight loss program, demonstrating the importance for future corporately sponsored weight loss programs to promote both components as part of their behavioral modification approaches.

Limitations of our study include the lack of a control group and the absence of dietary records. However, a 2012 systematic review and meta-analysis showed that no change in control group weight is typically observed in trials using control groups and that control groups receiving standard care typically lose ~ 1 kg more than control groups receiving no intervention [31]. Secondly, the retrospective self-report nature of outcomes and the lack of control group make it difficult to infer causality regarding the relationship between weight loss or changes in PA and improvements in QoL indices, and we have no record of these (potentially reduced) QoL indices before the start of the program. Further, self-report may be systematically biased in people with overweight and obesity as they may be more likely to under-report body weight [32]. Finally, the use of a non-standardized QoL measure is a limitation. However, a commercial weight loss program presents different challenges than research settings when designing questionnaires and surveys: 1) extensive baseline questionnaires (e.g., SF-36) greatly lengthen the application and enrolment process which affects completion rates and 2) the necessary trusting relationship with the participants has usually not yet been established during the application process, and psychosocial questions are therefore usually not well received and often lead to complaints to the human resources department.

## Conclusions

In the present study, we have demonstrated that among people who engaged with a web-based commercial weight loss program, short-term weight loss during the program was significantly associated with improvements in six indices of QoL (energy, mood, sleep, self-confidence, indigestion, and musculoskeletal pain) and that increases in PA during the program further enhance these benefits.

## Data Availability

The data that support the findings of this study are available from the corresponding author upon reasonable request.

## Abbreviations

BMI: Body mass index
CDC: Centers for Disease Control and Prevention
CI: Confidence interval
DREW: Dose-Response to Exercise in postmenopausal Women
IWQOL: Impact of Weight on Quality of Life
PA: Physical activity
PROMIS-29: 29-Item Patient-Reported Outcomes Measurement Information System
QoL: Quality of life
SD: Standard deviation
SMART: Specific, Measurable, Attainable, Realistic, Time-based
SF-36: 36-Item Short-Form Health Survey
